# Back to the Origin: *bla*_OXA-204_ and *bla*_NDM-1_ Genes in *Shewanella* spp. from a Tunisian River

**DOI:** 10.1128/spectrum.01605-22

**Published:** 2022-09-20

**Authors:** Sana Ncir, Agnese Lupo, Antoine Drapeau, Pierre Châtre, Meriem Souguir, Sana Azaiez, Jean-Yves Madec, Wejdene Mansour, Marisa Haenni

**Affiliations:** a Unité Antibiorésistance et Virulence Bactériennes, ANSES - Université de Lyon, Lyon, France; b Laboratoire de Biophysique métabolique et Pharmacologie Appliquée (LR12ES02), Faculté de Médecine Ibn Al Jazzar Sousse, Sousse, Tunisia; University of Pittsburgh School of Medicine

**Keywords:** carbapenem, *Shewanella xiamenensis*, *Shewanella bicestrii*, Tunisia, NDM-1, OXA-204, IS91, PER-1

## LETTER

The environmental bacteria *Xanthomonas* spp. and *Shewanella* spp. are progenitors of the *bla*_NDM-_ and *bla*_OXA-48-like_ carbapenemase genes, respectively (1, 2). The most likely hypothesis is that these genes have been mobilized in the environment on mobile genetic elements, and subsequently transferred to Gram-negative bacteria (GNB) of clinical importance. The pathogens (primarily Escherichia coli and Klebsiella pneumoniae) that acquired these carbapenemase genes then emerged as a major One Health issue and are currently reported not only in clinical settings but also in animals and the environment (3). In January 2019, two carbapenem-resistant (CP-R) *Shewanella* spp. were isolated from fresh water sampled at two different locations along the El Hammam Oued river in Sousse, Tunisia, as part of a pilot study on multidrug-resistant GNB in aquatic environments. This river collects urban treated wastewater but no hospital effluents. The two isolates resistant to ertapenem (MIC ≥32 mg/L) were whole-genome sequenced using short-read (NovaSeq-6000, Illumina) and long-read (MinION, Oxford Nanopore) technologies (all genomic sequences were deposited in DDBJ/EMBL/GenBank under the accession number PRJNA830355). Genomes were assembled with Unicycler and analyzed using tools of the Center for Genomic Epidemiology (https://www.genomicepidemiology.org/).

**FIG 1:**
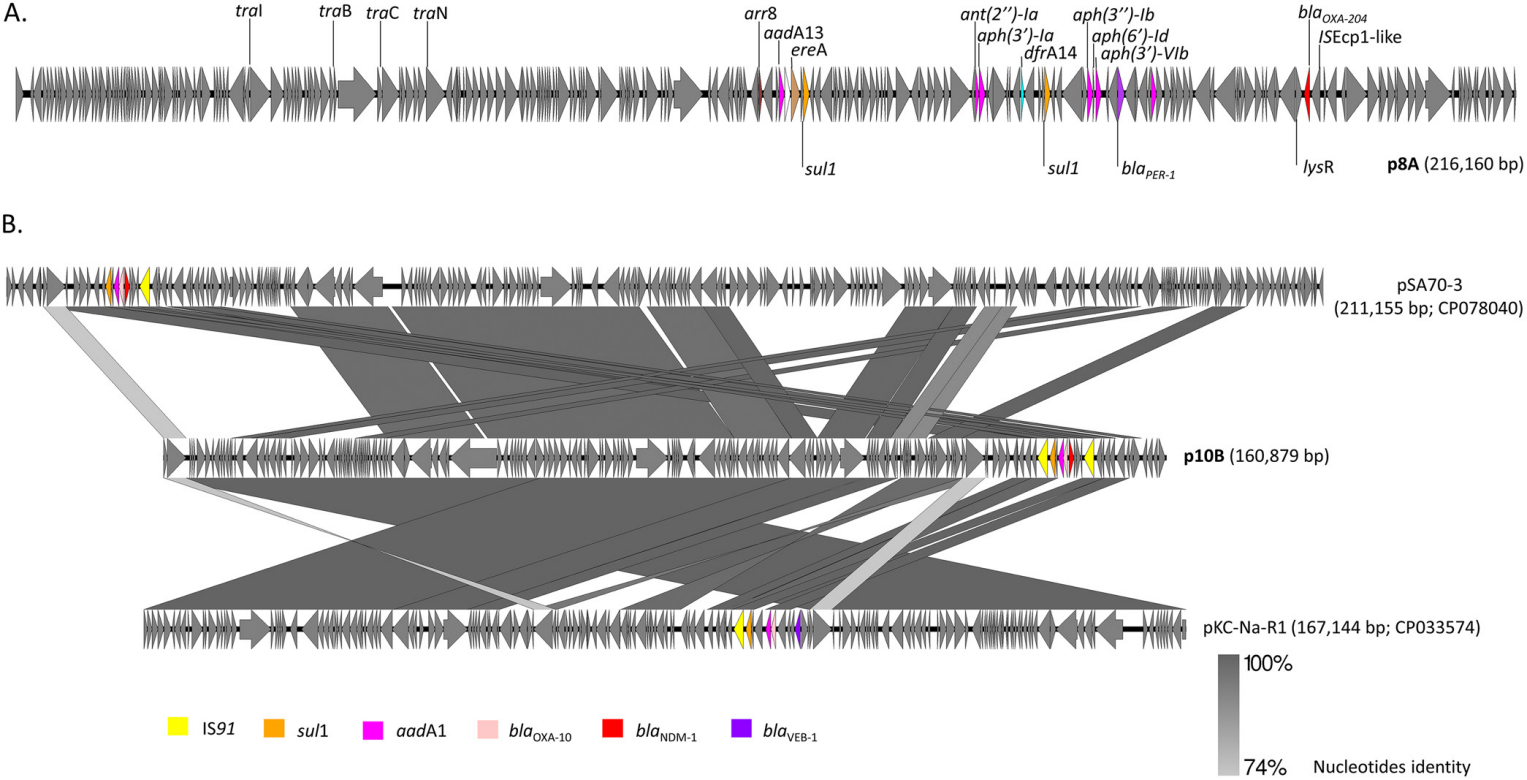
Schematic representation of the p8A plasmid (A). Comparative schematic representation of the p10B, pSA70-3 (CP078040), and pKC-Na-R1 (CP033574) plasmids. Genetic elements were analyzed by BLASTn and visualized using Easyfig 2.2.5_win. The window size used for BLASTn analysis was 2,000 bp.

*S. xiamenensis* 8A possessed a 4,959,567 bp chromosome and one nontypeable 216,160 bp plasmid (p8A) showing poor or fragmented homologies with published sequences. Except for *bla*_OXA-538_, all resistance genes—conferring resistances to beta-lactams, aminoglycosides, sulfonamides, trimethoprim, rifampicin and erythromycin—were located on p8A (Fig. 1A). Specifically, p8A carried the *bla*_PER-1_ extended-spectrum beta-lactamase (ESBL) and the *bla*_OXA-204_ carbapenemase genes. *bla*_OXA-204_ has already been reported in one *S. xiamenensis* strain from water in Portugal (4), flanked by the peptidase C15 and *lysR* gene as intrinsically found on the *Shewanella* spp. chromosome (5). Here, the *bla*_OXA-204_ gene was surrounded by an IS*Ecp1* insertion sequence and a truncated *lys*R gene, as reported in the K. pneumoniae KP49 isolated from a Tunisian patient (KP027886) (6). This indicates that *bla*_OXA-204_ in p8A is not intrinsic but has transited through another bacterial host before being reintegrated in *S. xiamenensis* p8A via a transposition event.

*Shewanella bicestrii* 10B possessed a 4,717,299 bp chromosome carrying a *bla*_OXA-48-like_ and the *aadA2* resistance genes. The strain additionally presented a nontypeable 160,879 bp plasmid (p10B) sharing conserved regions with the pSA70-3 and pKC-Na-R1 plasmids, respectively, isolated from *Shewanella putrefasciens* and Shewanella algae (Fig. 1B) (7). p10B presented genes conferring resistance to beta-lactams, aminoglycosides, sulfonamides, trimethoprim, chloramphenicol, quinolones, and rifampicin, but also to mercury and biocides. CP-R was conferred by the *bla*_NDM-1_ gene; *bla*_NDM-1_, together with the *bla*_OXA-10_, *sul1*, and *aadA1* genes, was surrounded by two IS*91*-like that might have mediated its insertion/excision (8). A similar genetic organization was observed in pSA70-3 with only the left IS*91*-like missing (7), while the element was more fragmented in pKC-Na-R1, where only the *sul1*-IS*91*-like and *aadA1*-*bla*_OXA-10_ genes were conserved upstream the *bla*_VEB-1_ ESBL gene.

Environmental bacteria are progenitors of carbapenemases (1, 2), but reacquisition of these genes by environmental bacteria is rarely reported. This study showed that the *bla*_OXA-204_ and *bla*_NDM-1_ genes, which are highly prevalent in Tunisian hospital settings and effluents (9, 10), can spread back to environmental bacteria. In both cases, resistance genes moved to a *Shewanella* spp. nontypeable plasmid after IS-mediated acquisition, a mechanism that might be more efficient than plasmid transfer for bacterial genera that are not closely related.
